# Empathy and nonattachment independently predict peer nominations of prosocial behavior of adolescents

**DOI:** 10.3389/fpsyg.2015.00263

**Published:** 2015-03-19

**Authors:** Baljinder K. Sahdra, Joseph Ciarrochi, Philip D. Parker, Sarah Marshall, Patrick Heaven

**Affiliations:** Institute for Positive Psychology and Education, Australian Catholic UniversityStrathfield, NSW, Australia

**Keywords:** prosocial behavior, empathy, nonattachment, multilevel poisson modeling, peer nominations

## Abstract

There is a plethora of research showing that empathy promotes prosocial behavior among young people. We examined a relatively new construct in the mindfulness literature, nonattachment, defined as a flexible way of relating to one's experiences without clinging to or suppressing them. We tested whether nonattachment could predict prosociality above and beyond empathy. Nonattachment implies high cognitive flexibility and sufficient mental resources to step out of excessive self-cherishing to be there for others in need. Multilevel Poisson models using a sample of 15-year olds (*N* = 1831) showed that empathy and nonattachment independently predicted prosocial behaviors of helpfulness and kindness, as judged by same-sex and opposite-sex peers, except for when boys nominated girls. The effects of nonattachment remained substantial in more conservative models including self-esteem and peer nominations of liking.

## Introduction

Prosociality can be expressed in many ways, such as through caring, comforting, cooperating, protecting, helping, sharing, donating, voting, and volunteering. The behaviors are considered prosocial to the extent that they are voluntary actions undertaken to promote the well-being of others or society at large, despite the personal cost to the actors (Eisenberg and Mussen, 1989; Eisenberg et al., 2006). The benefits for those at the receiving end of prosocial acts seem obvious, but prosocial behavior, especially when autonomously driven, can also promote the well-being of the helpers themselves (Weinstein and Ryan, 2010). In adolescents, in particular, potential personal benefits of prosocial behavior include higher educational aspirations and grade point averages, stronger intrinsic school work values, increased importance of community involvement, supportive peer relationships, reduced rates of school suspension, dropout and risky behaviors such as teen pregnancy, and higher overall life satisfaction (Moore and Allen, 1996; Johnson et al., 1998; Caprara et al., 2000; Markiewicz et al., 2001; Caprara and Steca, 2005). A large-scale review suggests that “giving” is one of the five “ways to well-being” (Aked et al., 2009). In short, prosociality is one of the key aspects of adolescents' personal and interpersonal flourishing.

What are the dispositional factors linked to prosocial behavior in adolescents? Answers to this question can inform basic research as well as interventions aimed at improving personal and interpersonal outcomes. This paper focuses on two individual difference variables hypothesized to underpin prosocial behavior: a well-studied construct of empathy and a relatively new construct of nonattachment from the growing field of mindfulness.

Empathy is defined as one's capacity to understand another person's perspectives and experience affective responses to another person's distressful condition (e.g., Eisenberg et al., 2006). This definition implies that empathy has two important components: a cognitive aspect involving perspective taking capacity, and an affective aspect involving emotional resonance with the distressful feelings of the person in need. There is plethora of research showing that empathy promotes prosocial behavior among young people as well as adults (e.g., Batson, 1991; Hoffman, 2000; Eisenberg et al., 2006). Empathy can help people orient their attention to other's feelings and needs, which in turn can motivate them to offer tangible help (Eisenberg, 1986; Batson, 1991; Hoffman, 2000). Since empathy is regarded as a key determinant of prosocial behavior, it is an ideal benchmark for examining the role of any new construct purported to be important for understanding prosociality.

The current study examines the role of a novel construct, nonattachment, in predicting adolescent prosocial behavior above and beyond the contributions of cognitive and affective empathy. Nonattachment is a relatively new construct in the mindfulness literature, with growing support for its personal and interpersonal benefits in clinical and non-clinical contexts (Sahdra et al., 2010; Allen, 2012; Epel et al., 2012; Sahdra and Shaver, 2013; Shonin et al., 2013a,b; Chang et al., 2014; Lamis and Dvorak, 2014). Mindfulness is often defined as attention to and awareness of the present experience (Brown and Ryan, 2003). Nonattachment is defined as a flexible, balanced way of relating to one's experiences without clinging to or suppressing them. As one might expect, nonattachment is positively related to mindfulness but empirically distinguishable from it (Sahdra et al., 2010). In a multidimensional assessment of mindfulness (using the Five Factor Mindfulness Questionnaire; Baer et al., 2008), nonattachment is most strongly linked to the non-reactivity aspect of mindfulness but remains empirically distinguishable from non-reactivity (Sahdra et al., unpublished manuscript).

Some of the earliest known discussions on nonattachment date back more than 2000 years in Eastern scholarly texts, where nonattachment is described as a remedy to the problem of getting stuck or “attached” to one's mistaken beliefs about the interdependent and ever-changing nature of self and others. In one text, for instance, “attachment” (Sanskrit: raga, upadana) is described as a “mental affliction that distorts the cognition of its object (such as the self or any other cherished object) by exaggerating its admirable qualities and screening out its disagreeable qualities” (Asanga, 4th–5th Century BCE/1950, as paraphrased by Sahdra et al., 2010, p. 116). This use of the word “attachment” implies fixation on mistakenly reified/solidified beliefs about oneself, other persons, social groups, attractive objects, the physical world, even life itself. In contrast, “nonattachment” (Sanskrit: viraga) denotes a release from the tendency to solidify personal beliefs as infallible reflections of a fixed knowable reality. Nonattachment to one's mental models (of the self, others, and the world at large) was thought by ancient Eastern scholars to promote objective perception of the interdependent and ever-changing aspects of reality, openness to undesirable facts of life, reduced selfishness, letting go of defensive grasping onto cherished beliefs, a genuine sense of connectedness to others, and generosity toward those in need (Sahdra et al., 2010).

It is important to clarify that lack of fixation on personal beliefs implied by nonattachment does not mean lack of connectedness to others or avoidance of closeness with others. Nonattachment differs conceptually from attachment theory's construct of avoidant attachment, which includes aversion to intimacy and interdependence in close relationships (Bowlby, 1969/1982; Ainsworth et al., 1978; Mikulincer and Shaver, 2007). Nonattachment is also conceptually distinct from anxious attachment, which involves intense concern with rejection and abandonment in close relationships. Indeed, nonattachment is inversely related to but empirically distinguishable from anxious and avoidant attachment (Sahdra et al., 2010). Further, it has been suggested that nonattachment may help soften the “us vs. them” divide that is often used to justify harming “them” to benefit “us” (Chang et al., 2014). Consistent with these empirical and theoretical perspectives, nonattachment predicts less closed-mindedness even after controlling for anxious and avoidant attachment (Sahdra and Shaver, 2013).

Nonattachment is also not the same as detachment or dissociation from one's own thoughts or feelings. Rather, it implies mental engagement with *both* desirable and undesirable aspects of one's experience *without* clinging to the desirable aspects or rejecting the undesirable ones. It is thought to counter a defeatist outlook of one's circumstances (Shonin et al., 2013a), and has been shown to buffer against self-harming in young adults by lowering their depressive symptoms (Lamis and Dvorak, 2014). Nonattachment has the subjective quality of ease and balance rather than a feeling of being mentally “trapped” and stuck or fixated on one's ideas about oneself, others, and the world. It is believed to free people from unhealthy fixations on self-cherishing or self-disparaging beliefs, and make it easy for them to care for others and take their perspective. Consistent with this theory, nonattachment has been shown to be inversely related to dissociative tendencies, and positively related to dispositional empathy and generosity in adults (Sahdra et al., 2010).

To our knowledge, there is no empirical research in the extant literature that directly examines the connections between nonattachment, empathy and prosocial behavior in young people, although some studies indirectly suggest that these variables might be related. Nonattachment implies a certain degree of self-awareness. In order to mentally “hold” one's experiences with nonattachment, one must be sufficiently aware of one's tendencies to mentally cling to desirable experiences or push away undesirable experiences. Prosocial children, compared to their less prosocial counterparts, tend to exhibit high levels of self-reflection and awareness of their family and personal beliefs and values (Hart and Fegley, 1995). They also exhibit high attentional regulation and constructive social skills (Eisenberg et al., 1996). Research also shows that a strong sense of efficacy in regulation of positive and negative affect is associated with empathy toward others' emotional experiences and prosocial behavior (Caprara and Steca, 2005). Nonattached individuals tend to show high dispositional empathy and less difficulty in emotion regulation (Sahdra et al., 2010), so they might also be more prosocial.

To help others means to give up self-enhancement for the moment and support other people's power, achievement, and personal success. Evidence from longitudinal research suggests that self-transcendence is an important determinant of prosociality (Caprara et al., 2012). Self-transcendence values of universalism and benevolence often conflict with self-enhancement values of power, achievement and personal success over that of others (Schwartz, 1992). The nonattached person is expected to let go of self-enhancement feelings and thoughts (“When pleasant experiences end, I am fine moving on to what comes next,” as an example item from the measure of nonattachment we used in this study; see Appendix A for all items). Furthermore, the nonattached person is expected to take joy in others' successes (“I can take joy in others' achievements without feeling envious,” as another scale item). In contrast, the attached person is expected to cling to personal joys that conflict with others' needs, and to avoid negative feelings, such as those that might arise from seeing others in distress or doing something socially risky to help another. Thus, we hypothesize that attachment, as defined here, will be linked to low levels of prosocial behavior.

If nonattachment entails a flexible use of executive control resources to attend to others' needs, it should be positively linked to the cognitive aspect of empathy. However, we hypothesize that nonattachment and empathy are distinct and will predict unique variance in prosociality. Theoretically, nonattachment focuses on the willingness to let go of personal joys that conflict with others, whereas empathy focuses on the ability to see things from another's viewpoint.

Any positive construct such as empathy or nonattachment may be linked to the commonly researched construct of self-esteem, which reflects positive regard for self. If so, then self-esteem would confound the relationships between nonattachment, empathy and prosociality. There is some evidence suggesting that self-esteem and prosociality might be positively related (Laible et al., 2004; Zuffianò et al., 2014). Self-esteem predicts increasing levels of social support over time (Marshall et al., 2014). Adolescents with high self-esteem also value prosocial means of achieving their goals (Smith et al., 1999). In an intervention study, participants of a school-based helper program who engaged in volunteering activities in school, compared to non-participants, showed a boost in self-esteem (Switzer et al., 1995). It is also possible that those with high self-esteem have high self-presentation concerns and exhibit prosocial behavior in order to enhance their positive public image (Baumeister, 1982), and not necessarily due to a genuine concern for others. But those who base their self-esteem on virtue, and not on gaining power over others, may also exhibit prosocial behavior (Crocker, 2002). Thus, there are multiple theories that would posit a link between self-esteem and prosociality. The present study sought to investigate the extent to which empathy and nonattachment predicted unique variance in prosociality, above and beyond self-esteem.

In the current study, we measured self-reported cognitive and affective empathy and nonattachment in a large sample of adolescents to test their relative contribution in predicting the participants' prosocial behavior as observed by their peers. We also measured self-reported self-esteem to control for high self-regard. Peer nominations are particularly useful in tapping into dispositional prosocial behavior across a variety of situations as opposed to situation-specific prosocial acts. This method also avoids the pitfalls of biased, socially desirable responding in self-report measures of prosociality. One possible issue with this method, however, is that peer nominations of prosocial behavior may reflect, at least in part, the peers' liking for the nominees regardless of the nominees' actual level of prosocial behavior (i.e., nominating individuals not because they are prosocial but because they are one's friend). We therefore obtained liking nominations as well to test whether the predictor variables of primary interest still explain variance in the prosociality outcome variables once the effects of liking are controlled for. If, however, prosociality causally precedes liking such that people like individuals who are prosocial, then using liking as a control variable would be a misspecification of the model. To deal with this issue, we ran models with and without liking as a control variable to examine the role of nonattachment and empathy in independently predicting prosocial nominations. Consistent with the empathy literature mentioned above, we expected cognitive and affective empathy to be related to prosociality nominations. Based on our theoretical discussion about nonattachment, we hypothesized that nonattachment would uniquely predict prosocial nominations above and beyond the contributions from other variables.

## Materials and methods

Participants were 1831 adolescents in Grade 10 from 16 high schools in New South Wales and Queensland, Australia. Of these, 923 were girls (age *M* = 15.63 years, *SD* = 0.43) and 908 were boys (age *M* = 15.67 years, *SD* = 0.43). The sample was part of the Australian Character Study, in which participants completed a battery of questionnaires. Paper-and-pencil questionnaires were administered using a similar procedure in all schools. Ethics approval was obtained from the University of Wollongong Human Research Ethics Committee (HE10/158) before data collection. The schools in this sample are fairly representative of the Australian population in terms of ethnicity, employment, and religious beliefs (Australian Bureau of Statistics, 2010). These schools had an average score of socioeconomic index of 1026 (*SD* = 43), which is comparable to the average of 1000 provided by the Australian government for all schools across Australia (http://www.bit.ly/1mJK7KC). Further details about the sample are reported elsewhere (Parker et al., 2014; Marshall et al., 2015). For the purposes of this paper, we focus on the following measurements:

*Empathy* was measured using the 20-item Basic Empathy Scale that has been well-validated in adolescents and has been consistently shown to have a two-factor structure, with nine items measuring cognitive empathy and 11 measuring affective empathy (Jolliffe and Farrington, 2006; Albiero et al., 2009). An example item of cognitive empathy is, “I can often understand how people are feeling even before they tell me;” and that of affective empathy is, “I get caught up in other people's feelings easily.” Participants used a scale ranging from 1 to 5 to indicate the degree to which the items applied to them. The alpha coefficient for cognitive empathy items was 0.69 and that for affective empathy items was 0.73.

*Nonattachment* was assessed using the nine-item Nonattachment Scale or NAS-7 (Elphinstone et al., unpublished manuscript), which is a shorter version of the original 30-item Nonattachment Scale (Sahdra et al., 2010). The measure consists of a six-point Likert scale on which participants rate their agreement with the seven items (as all items in Appendix A). NAS-7 has recently been shown to have excellent psychometric properties in American and Australian adult samples (Elphinstone et al., unpublished manuscript). Higher NAS-7 scores were associated with greater mindfulness, autonomous regulation, self-actualization, well-being, less importance placed on extrinsic aspirations and consumer materialism, and lower levels of depression. The correlations of NAS-7 with the measures of these constructs were comparable to the correlations of the NAS-30 with the same measures. NAS-7 measured nonattachment was also empirically distinct from experiential avoidance. In a separate study using a nationally representative American adults sample, nonattachment, as measured by either the 30-item original measure or the short seven-item measure, was found to be empirically distinct from the non-reactivity aspect of mindfulness, further validating the short measure (Sahdra et al., unpublished manuscript). To test the fit of NAS-7 in our adolescent sample, we conducted a confirmatory factor analysis of the seven items using the lavaan package (Rosseel, 2012) in R (R Core Team, 2013). The seven items yielded a good fit, χ^2^(14) = 155.28, *p* < 0.001, CFI = 0.96, TLI = 0.94, RMSEA = 0.07, SRMR = 0.03. The omega coefficient of the internal structure of the scale was satisfactory, 0.82. The alpha coefficient was also 0.82.

*Self-esteem* was measured using the Rosenberg 10-item scale (Rosenberg, 1979) with a binary response style of “yes” or “no” for each item (which has been validated by Marshall et al., 2014, in their study on adolescents). Sample items include: “I feel that I am a person of value – equal to most other kids my age,” and “Generally, I feel satisfied with myself.” The alpha coefficient of internal consistency was 0.86.

*Peer nominations of prosociality and liking* were obtained using items identical to the ones used by Ciarrochi and Heaven (2009), which were based on the peer-rating measure validated by Pulkkinen et al. (1999). Participants were asked to nominate same-sex and opposite-sex peers that “are ready to lend a helping hand when they see someone in need of that” and “are often kind and friendly to others,” and peers that they “like the most.” Participants were asked to nominate up to three peers of each gender in each category. The peer nominations data were coded such that each participant received separate scores representing the counts of nominations she or he received from same-sex and opposite-sex peers for each of the two prosociality items and the liking item. We analyzed helpfulness and kindness variables separately because, as detailed below, these two variables tapped into different aspects of prosociality.

## Results

In keeping with the guidelines in the American Psychological Association's (APA's) *Publication Manual* (APA, 2010), we employed an estimation-driven approach to finding plausible population parameters. In contrast to *p*-values, which can vary dramatically from one replication to another of the same study, confidence intervals (CIs) of effect sizes are far more informative, especially in the context of a single study (Cumming, 2013). For instance, a 95% CI is an 83% prediction interval for the effect size estimate of a replication study, and a value close to the center of CI is about seven times as likely to be the population parameter as is a value near the limit of the 95% CI (Cumming and Maillardet, 2006). The statistical program R (R Core Team, 2013) was used to calculate all point estimates and CIs reported in this paper.

### Peer nominations of prosociality

**Figure 1:**
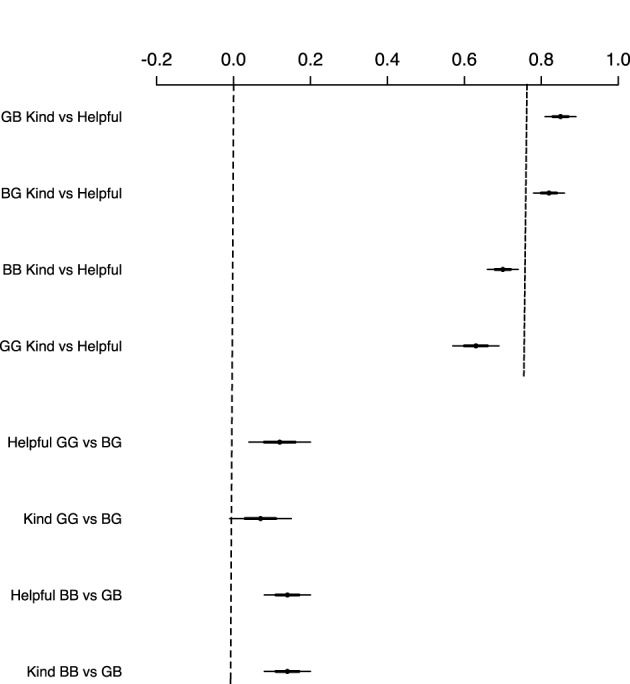
**Correlations of same-sex and opposite-sex nominations of helpfulness and kindness, and BCa bootstrapped 90% (darker lines) and 95% (lighter lines) confidence intervals (CIs)**. Kind: counts of peer nominations for being “often kind and friendly toward others;” helpful: counts of peer nominations for being “ready to lend a helping hand when they see someone in need of that;” GB: girls nominating boys; BG: boys nominating girls; BB: boys nominating boys; GG: girls nominating girls. A vertical line on the top right of the figure at around 0.75 mark does not cross any of the CIs, showing that the same-sex and opposite-sex correlations flanking on the two sides of the line appear to be reliably different from each other.

Point estimates and 95% CIs for inter-correlations between all variables of the study were calculated using the *bias-corrected-and-accelerated* (BCa) bootstrap procedure implemented in the bootES package (Gerlanc and Kirby, 2013; Kirby and Gerlanc, 2013) in R. Parametric CIs are not robust to violations of normality (Kelley, 2005). Bootstrapping is a much better approach because it makes no assumptions about the shape of distributions of the sample statistic. Tables 1, 2 contain the BCa bootstrapped estimates and 95% CIs of the inter-correlations of all variables for boys and girls, respectively. Figure 1 visually depicts the inter-correlations of the key outcome variables of peer nominations of kindness and helpfulness, and includes both 90% (darker lines) and 95% (lighter lines) CIs.

**Table 1:** **Bias-corrected-and-accelerated bootstrapped estimates and 95% confidence intervals of inter-correlations between all variables for boys**.

	**BB helpful**	**BB kind**	**GB helpful**	**GB kind**	**BB liking**	**GB liking**	**Cog. empathy**	**Aff. empathy**	**Self-esteem**
BB kind	0.70 (0.66–0.75)								
GB helpful	0.14 (0.08–0.20)	0.14 (0.08–0.20)							
GB kind	0.10 (0.04–0.15)	0.14 (0.08–0.20)	0.85 (0.81–0.89)						
BB liking	0.54 (0.49–0.59)	0.64 (0.59–0.68)	0.08 (0.02–0.14)	0.07 (0.01–0.14)					
GB liking	0.09 (0.03–0.15)	0.12 (0.06–0.18)	0.82 (0.78–0.86)	0.87 (0.84–0.90)	0.08 (0.02–0.15)				
Cog. empathy	0.14 (0.09–0.19)	0.14 (0.09–0.20)	0.16 (0.10–0.21)	0.15 (0.09–0.21)	0.13 (0.07–0.19)	0.14 (0.08–0.20)			
Aff. empathy	0.11 (0.05–0.17)	0.12 (0.06–0.19)	0.11 (0.05–0.16)	0.09 (0.03–0.15)	0.08 (0.02–0.15)	0.09 (0.03–0.15)	0.43 (0.38–0.49)		
Self-esteem	0.11 (0.06–0.17)	0.10 (0.04–0.16)	0.02 (−0.04–0.08)	0.05 (−0.01–0.10)	0.12 (0.07–0.18)	0.04 (−0.02–0.09)	0.004 (−0.07–0.08)	−0.14 (−0.20 to −0.06)	
Nonattachment	0.14 (0.08–0.19)	0.12 (0.06–0.18)	0.09 (0.04–0.15)	0.11 (0.05–0.17)	0.07 (0.01–0.13)	0.06 (−0.01–0.12)	0.29 (0.21–0.36)	−0.01 (−0.08–0.07)	0.37 (0.30–0.43)

*Kind: counts of peer nominations for being “often kind and friendly toward others;” helpful: counts of peer nominations for being “ready to lend a helping hand when they see someone in need of that;” liking: counts of peer nominations for being “liked the most.” BB: boys nominating boys; GB: girls nominating boys; Cog. empathy: cognitive empathy; Aff. empathy: affective empathy*.

**Table 2:** **Bias-corrected-and-accelerated bootstrapped estimates and 95% confidence intervals of inter-correlations between all variables for girls**.

	**BG helpful**	**BG kind**	**GG helpful**	**GG kind**	**BG liking**	**GG liking**	**Cog. empathy**	**Aff. empathy**	**Self-esteem**
BG kind	0.82 (0.78–0.85)								
GG helpful	0.12 (0.06–0.20)	0.07 (0.01–0.14)							
GG kind	0.08 (0.01–0.15)	0.07 (0.00–0.14)	0.63 (0.57–0.68)						
BG liking	0.81 (0.78–0.85)	0.88 (0.85–0.90)	0.03 (-0.03–0.10)	0.02 (-0.04–0.09)					
GG liking	0.02 (−0.04–0.09)	−0.003 (−0.07–0.06)	0.48 (0.42–0.54)	0.61 (0.56–0.66)	−0.02 (−0.08–0.05)				
Cog. empathy	0.05 (−0.01–0.11)	0.05 (−0.01–0.10)	0.09 (0.03–0.15)	0.08 (0.02–0.15)	0.03 (−0.03–0.08)	0.04 (−0.03–0.10)			
Aff. empathy	0.05 (−0.02–0.11)	0.03 (−0.04–0.09)	0.10 (0.04–0.16)	0.11 (0.05–0.17)	0.03 (−0.04–0.10)	0.09 (0.02–0.15)	0.47 (0.42–0.52)		
Self-esteem	−0.004 (−0.06–0.05)	−0.01 (−0.07–0.05)	0.14 (0.08–0.20)	0.13 (0.07–0.19)	−0.03 (−0.09–0.04)	0.06 (−0.01–0.12)	−0.02 (−0.08–0.05)	−0.13 (−0.20 to −0.06)	
Nonattachment	0.03 (−0.03–0.09)	0.03 (−0.04–0.09)	0.17 (0.11–0.23)	0.16 (0.11–0.23)	0.01 (−0.06–0.07)	0.07 (0.00–0.13)	0.20 (0.13–0.27)	0.02 (−0.05–0.10)	0.50 (0.44–0.54)

*Kind: counts of peer nominations for being “often kind and friendly toward others;” helpful: counts of peer nominations for being “ready to lend a helping hand when they see someone in need of that;” liking: counts of peer nominations for being “liked the most.” BG: boys nominating girls; GG: girls nominating girls; Cog. empathy: cognitive empathy; Aff. empathy: affective empathy*.

As shown in the top half of Figure 1, the correlations between peer nominations for helpfulness and kindness were generally high, ranging from 0.63, 95% CI (0.57–0.68) to 0.85 (0.80–0.89), suggesting that those who were nominated as kind by their peers tended to be nominated as helpful as well by their peers. However, the same-sex (e.g., boys nominating boys on helpfulness correlated with boys nominating boys on kindness) and opposite-sex correlations (e.g., boys nominating girls on helpfulness correlated with boys nominating girls on kindness) were different from each other, as is clearly depicted by a dashed line separating the two sets of correlations in the top right of Figure 1. The same-sex correlations flanking on the left of the dashed line were lower than the opposite-sex correlations on the right, suggesting that participants discriminated between helpfulness and kindness dimensions of prosociality more so when making same-sex nominations than when making opposite-sex nominations.

Further, as shown in the bottom left of Figure 1, the correlations between same-sex and opposite-sex nominations for any given prosociality variable (e.g., girls nominating girls on helpfulness correlated with boys rating girls on helpfulness) were very small or negligible, suggesting that boys and girls nominated different peers whom they judged as helpful and kind. Taken together, the results depicted in Figure 1 suggest that the two measures of prosociality, kindness and helpfulness, tapped into distinct aspects of prosocial behaviors.

### Peer nominations of liking

The BCa bootstrapped estimate of the correlation between boys nominating boys and girls nominating boys on liking was 0.08 95% CI (0.02–0.15), and the correlation between boys nominating girls and girls nominating girls was 0.02 (−0.07–0.05), suggesting that boys and girls judged different peers as likable. Regarding the correlation of liking with our key predictor variables of empathy and nonattachment: as shown in Tables 1, 2, the BCa bootstrapped correlation estimates ranged from 0.04 (−0.02–0.09) to 0.14 (0.08–0.20) for boys, and from −0.02 (−0.08–0.05) to 0.09 (0.02–0.15) for girls. These negligible or small correlations suggest that adolescents who rated high on empathy or nonattachment were not necessarily the most liked by their peers. With respect to the correlations between liking and prosocial nominations, the estimates ranged from 0.54 (0.49–0.59) to 0.87 (0.84–0.90) for boys, and from 0.48 (0.42–0.54) to 0.88 (0.85–0.90), suggesting that prosocial individuals were also highly likable peers. Therefore, liking and prosocial nominations shared substantial variance to justify running our main models with and without using liking as a control variable to examine the relative contributions of empathy and nonattachment in predicting prosocial nominations.

### Self-esteem

As reported in Tables 1, 2, self-esteem was positively related to same-sex nominations of kindness and helpfulness. Self-esteem was negatively related to affective empathy but positively related to nonattachment among both boys and girls. Since self-esteem shared some variance with the predictor and outcome variables, it was reasonable to run models with and without using self-esteem as a covariate.

### Predicting prosociality from empathy and nonattachment

As reported in Tables 1, 2, nonattachment showed a small correlation with cognitive empathy, 0.29 95% CI (0.21–0.36) for boys and 0.20 (0.13–0.27) for girls, whereas almost zero correlation with affective empathy. All the predictor measures were standardized and entered in models to see their relative contribution in explaining the variance in the counts of peer nominations–how often the person has been nominated as kind or helpful by same-sex and opposite-sex peers. Poisson regression models are most suitable for analyzing count data (Cameron and Trivedi, 2013). However, Poisson models are subject to overdispersion, that is, having higher data-level variation than would be predicted by the model, because these models do not have variance parameters to capture the variation in the data. To deal with this issue, we used a multilevel Poisson modeling in which overdispersion was modeled using a data-level variance component (Gelman and Hill, 2007). Multilevel modeling also allowed us to account for class- and school-level variability.

**Figure 2:**
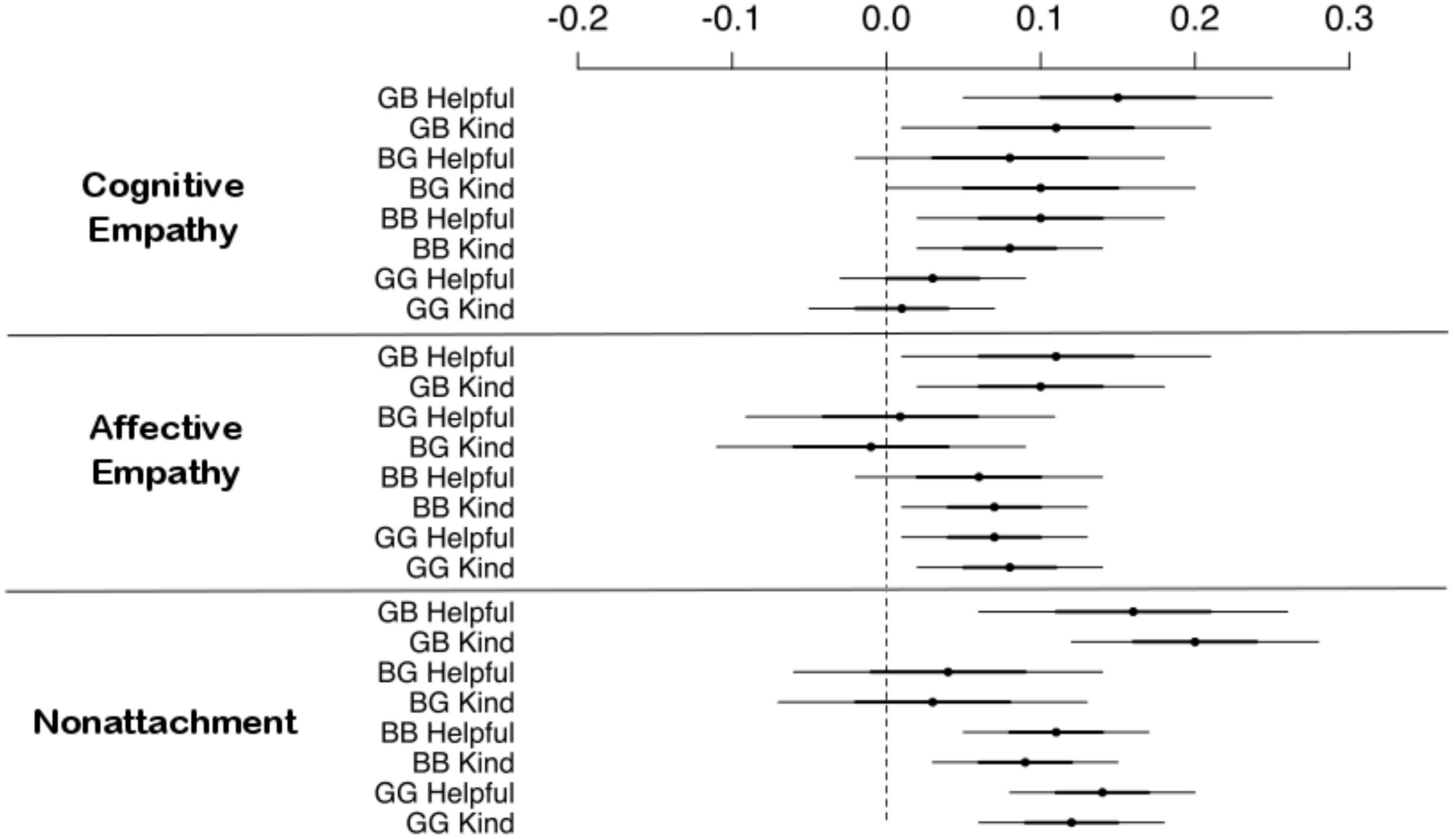
**Fixed effects estimates, and 90% (darker lines) and 95% (lighter lines) confidence intervals from multilevel Poisson models containing self-reported cognitive and affective empathy and nonattachment as predictors of prosocial peer nominations (without controlling for liking and self-esteem)**. GB: girls nominating boys; BG: boys nominating girls; BB: boys nominating boys; GG: girls nominating girls.

We ran a series of three-level Poisson regression models in which individual students were nested within classes, and classes within schools. The lme4 package (Bates et al., 2014) in R was used to conduct separate Poisson multilevel models for each of the two peer nominations counts separately for same-sex and opposite-sex nominations. We chose varying intercepts and constant slopes models because allowing the slopes to vary did not improve the model for any of the outcome variables (*p* > 0.10 for all likelihood ratio tests of model comparisons). To calculate CIs for the coefficients from the multilevel Poisson models, we used the profile method, which computes a likelihood profile and yields upper and lower cut-offs based on the likelihood ratio test relative to the “complete” likelihood. Table 3 contains the fixed effects coefficients and 95% CIs for cognitive and affective empathy and nonattachment from the multilevel Poisson regression models. Figure 2 contains a visual comparison of the pattern of fixed effects of the three predictors. It includes 90% CIs (darker lines) in addition to the longer 95% CIs (lighter lines).

**Table 3:** **Fixed effects parameters and 95% confidence intervals from multilevel Poisson regression models of opposite-sex and same-sex peer nominations, without and with controlling for liking and self-esteem**.

	**Cognitive empathy**	**Affective empathy**	**Nonattachment**
Girls nominating boys			
*Ready to lend a helping hand*	0.15^b^ (0.04–0.25)	0.11^a^ (0.02–0.21)	0.16^c^ (0.07–0.25)
Controlling for liking and SE	0.11^a^ (0.02–0.20)	0.05 (−0.03–0.13)	0.11^b^ (0.03–0.20)
*Act kind and friendly to others*	0.10^a^ (0.01–0.21)	0.10^a^ (0.01–0.18)	0.20^c^ (0.12–0.29)
Controlling for liking and SE	0.08 (−0.01–0.16)	0.02 (−0.05–0.09)	0.12^b^ (0.04–0.20)
Boys nominating girl			
*Ready to lend a helping hand*	0.08 (−0.02–0.19)	0.01 (−0.10–0.11)	0.04 (−0.06–0.14)
Controlling for liking and SE	0.04 (−0.05–0.13)	0.05 (−0.04–0.13)	0.01 (−0.08–0.11)
*Act kind and friendly to others*	0.10^a^ (0.00–0.20)	−0.01 (−0.001–0.09)	0.03 (−0.06–0.13)
Controlling for liking and SE	0.06 (−0.02–0.14)	0.01 (−0.06–0.09)	0.03 (−0.05–0.11)
Boys nominating boys			
*Ready to lend a helping hand*	0.10^a^ (0.02–0.18)	0.06 (−0.01–0.13)	0.11^c^ (0.04–0.17)
Controlling for liking and SE	0.05 (−0.02–0.12)	0.05 (−0.01–0.11)	0.09^b^ (0.03–0.15)
*Act kind and friendly to others*	0.08^a^ (0.01–0.14)	0.07^a^ (0.01–0.13)	0.09^b^ (0.03–0.14)
Controlling for liking and SE	0.03 (−0.02–0.09)	0.05 (−0.002–0.10)	0.06^a^ (0.01–0.12)
Girls nominating girls			
*Ready to lend a helping hand*	0.03 (−0.03–0.09)	0.07^a^ (0.01–0.13)	0.14^c^ (0.00–0.20)
Controlling for liking and SE	0.04 (−0.02–0.10)	0.04 (−0.02–0.10)	0.09^b^ (0.02–0.15)
*Act kind and friendly to others*	0.01 (−0.04–0.07)	0.08^b^ (0.02–0.13)	0.12^c^ (0.07–0.17)
Controlling for liking and SE	0.01 (−0.04–0.06)	0.04 (−0.01–0.09)	0.07^b^ (0.02–0.13)

*These effects from Poisson models are on the logarithmic scale, so an effect of 0.20, for instance, corresponds to a multiplicative effect of exp (0.20) = 1.22, or a 22% increase in the probability of being nominated by a peer with each standard deviation increase in the predictor variable. SE: self-esteem*.

a p < 0.05;

b p < 0.01;

c *p < 0.001*.

### Opposite-sex nominations

The results for opposite-sex nominations are reported in the top half of Table 3 and the top four lines in each of the three sections of Figure 2. When girls nominated boys, cognitive empathy, affective empathy and nonattachment independently predicted helpfulness and kindness ratings. The magnitude of the fixed effects coefficients ranged from 0.10 95% CI (0.01–0.18) to 0.20 (0.12–0.29). As in any Poisson model, all effects from our models are on the logarithmic scale. A fixed effect coefficient of 0.20, therefore, corresponds to an effect of exp(0.20) = 1.22, or a 22% increase in the probability of being nominated by a peer with each standard deviation increase in self-reported nonattachment. Table 3 further shows that adding self-esteem and liking nominations as control variables decreased the magnitude of all effects, as one would expect by increasing the number of correlated predictors. The fixed effects of affective empathy were all but eliminated. The effect of cognitive empathy on helpfulness remained substantial but its effect on kindness nominations was eliminated. However, the fixed effects of nonattachment on both helpfulness and kindness remained substantial even in these most conservative models. In short, cognitive empathy and nonattachment were distinctive predictors of boys' prosociality as observed by girls.

When boys nominate girls, neither affective empathy nor nonattachment were linked to any of the two prosociality variables. Cognitive empathy's fixed effect coefficient for kindness nominations was 0.10 (0.001–0.20), but this effect was eliminated in the model including self-esteem and liking as control variables. The results show that when boys nominated girls, they did not discriminate between those high or low in empathy or nonattachment while judging them as prosocial.

### Same-sex nominations

The bottom half of Table 3 and the bottom four lines in each of the three sections of Figure 2 show the results for same-sex nominations. When boys nominated boys, cognitive empathy and nonattachment were more reliable predictors than affective empathy in predicting helpfulness nominations. All three predictors were important in predicting kindness nominations. However, when self-esteem and liking were added to the models, the effects of cognitive and affective empathy were eliminated while nonattachment continued to explain substantial variance in kindness and helpfulness nominations. Similar to the findings of girls nominating boys, boys nominated other boys exhibiting high nonattachment as more prosocial.

When girls nominated girls, affective empathy and nonattachment were more reliable than cognitive empathy in predicting helpfulness and kindness nominations. However, when liking and self-esteem were added as covariates, the effects of affective empathy were eliminated whereas those of nonattachment continued to be substantial. Here again, as in other models of girls nominating boys and boys nominating boys, girls judged other girls who were high in nonattachment as kind and helpful.

## Discussion

We sought to test whether a relatively new construct in the mindfulness literature, nonattachment, defined as a flexible, balanced way of relating to one's experiences without clinging to or suppressing them, was linked to dispositional prosociality among adolescents above and beyond affective and cognitive empathy, self-esteem, and likeability. Prosociality was measured through peer nominations of individuals who were deemed as helpful and kind toward others. The two measures of prosociality appeared to be tapping into distinct aspects of prosociality because same-sex and opposite-sex peers judged each other differently on helpfulness and kindness. Cognitive and affective empathy and nonattachment were used as predictors in multilevel Poisson models to examine their relative contribution in explaining the variance in counts of peer nominations. We ran models with and without using peer nominations of liking and self-esteem as control variables to test whether removing variance associated with these variables made a difference in the pattern of results of models predicting peer nominations using the main predictors of empathy and nonattachment. In all models excluding the covariates, empathy and nonattachment were independent predictors of peer nominations, except when boys nominated girls. Cognitive empathy mattered more than affective empathy when girls and boys nominated boys whereas affective empathy mattered more than cognitive empathy when girls nominated girls. However, when self-esteem and liking were added to the models, neither cognitive nor affective empathy was important, suggesting that the effects of self-reported empathy are largely attributable to the control variables. However, nonattachment continued to explain substantial variance in prosocial nominations in these highly conservative models. Although self-reported self-esteem was substantially correlated with self-reported nonattachment, the links between nonattachment and different peer nominations cannot be attributed to self-esteem.

Prosocial individuals were generally highly liked by their same-sex and opposite-sex peers. However, boys and girls nominated different people as likable. They also nominated different people as prosocial, as reflected in negligible to small correlations between their nominations. It is possible that boys and girls may be picking up on different aspects of prosociality in different people, but one commonality in the individuals they choose seems to that those individuals are high in nonattachment.

It is not clear, however, why boys do not nominate girls scoring high on nonattachment as prosocial. The lack of relation between girls' nonattachment and boys' nominations of girls' prosociality could be either due to lack of demonstration of prosociality by girls to boys or lack of skill on the boys' part in detecting girls' prosocial behaviors or some other unknown reason. Future studies are needed to shed light on this asymmetry of why boys do not pick girls but they do pick boys scoring high on nonattachment as prosocial, whereas girls nominate other girls as well as boys scoring high on nonattachment as kind and helpful.

Future studies should also examine biological underpinnings of nonattachment and prosociality. The concept of nonattachment seems similar to the concept of cognitive flexibility in the executive functioning (EF) literature. EF is postulated to be a multi-faceted system of cognitive processes essential for higher order mental functions, including complex social information processing. These EF processes include, but are not limited to, working memory, attention, shifting, response inhibition, cognitive flexibility, and impulse control (Anderson, 2002; Best and Miller, 2010; Diamond, 2013). The EF processes are mediated mainly by prefrontal cortex and modulated by dopaminergic, noradrenergic, serotonergic, and cholinergic neurotransmitter systems, which allow the organism to flexibly adapt to the changing environment (Logue and Gould, 2014). The cognitive flexibility aspect of EF, in particular, may be related to nonattachment. Cognitive flexibility is an umbrella term including creatively thinking “outside the box,” the ability to take multiple perspectives on any given subject, and adapting to changing circumstances relatively quickly (Diamond, 2013). Recent evidence shows that cognitive flexibility predicts social understanding (theory of mind) in middle childhood (7–12 years) over and above the effects of age, vocabulary, working memory and inhibition (Bock et al., 2014). Cognitive flexibility is thought to be relatively mature by 12 years of age (Anderson, 2002). Given that cognitive flexibility and higher order social information processing are linked beyond their emergence in early childhood, and given our data showing positive relations between nonattachment and prosociality-relevant measures (empathy and peer nominations of kindness and helpfulness), examining the connection between cognitive flexibility and nonattachment might be a promising line of inquiry for future research.

One limitation of the present study is the cross-sectional nature of our data precluding testing of causal directions. This weakness can be remedied in future longitudinal and experimental studies examining nonattachment and prosociality over time. The effect sizes observed in our data ranged from a 7% to about 20% increase in probability of being nominated by a peer with each standard deviation increase in self-reported nonattachment. These effects sizes are comparable to the effect sizes in other multi-method studies, such as those linking negative affectivity and heart disease, the triple marker screening and Down's syndrome, self-reported hopelessness and subsequent suicide, extraversion scores and success in sales career, and familial social support and lower blood pressure (Meyer et al., 2001).

Consistent with previous literature, empathy was a reliable predictor of peer nominations of prosociality in the current study. However, relative to the effects of nonattachment, empathy did not fare well in predicting peer nominations when self-esteem and peer liking nominations were added to the models. In the models including all variables, with each standard deviation increase in empathy, there was very low probability (less than 5%) of being nominated as prosocial by a same-sex or opposite-sex peer. The apparent inconsistency between these results and previous research linking empathy and prosociality might be due to several reasons. We measured prosociality through peer nominations whereas most of the research on prosociality in adolescents typically uses self-report measures, which are arguably subject to self-serving and socially desirable responding. Further, in our full models, the inclusion of self-esteem and liking may have had a disproportionate effect on dampening the coefficients of empathy, compared to nonattachment, because of slightly stronger correlations of empathy with the covariates. Finally, self-esteem might be a cause of empathy, and not just a covariate. In other words, it is possible that self-esteem might cause empathy, which in turn, might cause prosocial behavior, independent of the effects of nonattachment on prosociality. A cross-sectional study cannot provide conclusive evidence regarding these issues, so we caution against any premature conclusions about empathy being any less important than nonattachment for understanding or promoting prosocial behavior among adolescents.

A notable strength of our study is that it is the first of its kind to pit empathy, which is generally regarded as a benchmark variable in understanding prosocial behavior, against an unseemly candidate of nonattachment, which at first can be easily mistaken as meaning not-attaching-to-others, thus an antithesis of empathy. Nonattachment is a very positive construct, implying high cognitive flexibility and sufficient mental resources to step out of excessive self-cherishing to better connect with others and be there for them in their time of need. We tested whether empathy and nonattachment could independently predict prosocial behavior as judged by peers. To make the test even more conservative, we controlled for peer-nominated liking and self-esteem. Still, nonattachment explained substantial variance in prosociality (except when boys nominated girls) independently of empathy. The results suggest that nonattachment is important for prosociality, but it would be wrong to conclude from our study that empathy is not at all important. Socio-emotional learning interventions harnessing the power of both empathy and nonattachment may well benefit young people more than either approach alone.

### Conflict of interest statement

The authors declare that the research was conducted in the absence of any commercial or financial relationships that could be construed as a potential conflict of interest.

## Appendix A

### The 7-item nonattachment scale (NAS-7)

(Elphinstone et al., unpublished manuscript)

To help us understand your general approach to life and your views about yourself, others, and life in general, tell us the extent to which the following statements reflect your experiences **at this point in your life**. Select a number from 1 to 6 on the scale provided with each statement to rate the extent to which you agree with it.

Please answer according to what **really reflects** your experience rather than what you think your experience should be.

**Table:**

**1**	**2**	**3**	**4**	**5**	**6**
Disagree	Disagree	Disagree	Agree	Agree	Agree
Strongly	Moderately	Slightly	Slightly	Moderately	Strongly

1. I can let go of regrets and feelings of dissatisfaction about the past.
2. I can enjoy pleasant experiences without needing them to last forever.
3. I view the problems that enter my life as things/issues to work on rather than reasons for becoming disheartened or demoralized.
4. I can enjoy my family and friends without feeling I need to hang on to them.
5. I can take joy in others' achievements without feeling envious.
6. I do not get “hung up” on wanting an “ideal” or “perfect” life.
7. When pleasant experiences end, I am fine moving on to what comes next.

Note. The seven items yielded a respectable fit in the current study, χ^2^(14) = 155.28, *p* < 0.001, CFI = 0.96, TLI = 0.94, RMSEA = 0.07, SRMR = 0.03. The omega coefficient of the internal structure of the scale was acceptable, 0.82, and the alpha coefficient was also 0.82.
